# Association between social network and dietary variety among community-dwelling older adults

**DOI:** 10.1017/S1368980023001325

**Published:** 2023-11

**Authors:** Miyuki Yokoro, Naoto Otaki, Tomomi Imamura, Norikazu Tanino, Keisuke Fukuo

**Affiliations:** 1 Department of Dietary Life and Food Sciences, Junior College Division, Mukogawa Women’s University, 6-46 Ikebiraki-cho, Nishinomiya, Hyogo 663-8558, Japan; 2 Research Institute for Nutrition Sciences, Mukogawa Women’s University, 6-46 Ikebiraki-cho, Nishinomiya, Hyogo 663-8558, Japan; 3 Department of Food Sciences and Nutrition, School of Food Sciences and Nutrition, Mukogawa Women’s University, 6-46 Ikebiraki-cho, Nishinomiya, Hyogo 663-8558, Japan; 4 Department of Innovative Food Sciences, School of Food Sciences and Nutrition, Mukogawa Women’s University, 6-46 Ikebiraki-cho, Nishinomiya, Hyogo 663-8558, Japan

**Keywords:** Social network, Dietary variety, Social isolation, Older adults

## Abstract

**Objective:**

Social networks are critical social health factors for older adults. This study examined the association between social networks and dietary variety among community-dwelling older adults.

**Design:**

A cross-sectional study, using the dietary variety score (DVS) developed for older Japanese people to assess dietary variety and the Lubben Social Network Scale (LSNS-6) to assess social networks.

**Setting:**

N City, H Prefecture, Japan.

**Participants:**

Community-dwelling older adults aged ≥ 65 years (*n* 1229).

**Results:**

The LSNS-6 score in the low DVS group was lower than that in the middle and high DVS groups (12·2 ± 5·6 *v*. 13·4 ± 5·4 and 14·4 ± 5·7, *P* < 0·001). The population of social isolation (LSNS-6, < 12) in the low DVS group was higher than that in the middle and high DVS groups (43·5 % *v*. 35·8 % and 31·0 %, *P* = 0·005). Multivariate linear regression analysis showed that the LSNS-6 score was positively correlated with DVS (standardised coefficient, 0·092; *P* = 0·002). Social isolation was also significantly associated with a low DVS in the multivariate-adjusted logistic analysis model (OR, 1·30; 95 % CI 1·00, 1·68; *P* = 0·048). Stratified analysis results revealed the LSNS-6 and DVS were significantly associated in participants with the following characteristics: younger age (< 75 years), women and those living with someone.

**Conclusions:**

Social networks were associated with dietary variety; social isolation was related to poor dietary variety among community-dwelling older adults. An association between social networks and dietary variety was observed among young-old older adults, women and those living with someone.

A social network refers to the structural aspects of social ties, such as the size and frequency of contact with others, and is considered an indicator of social integration and social engagement^(1,2)^. Small social networks result in social isolation^(3)^. Social isolation can lead to malnutrition, depression, lower cognitive function and frailty among older adults^(4–7)^. Furthermore, social isolation increased the risks of CVD and mortality^(8,9)^. Research suggests that social networks could be related to health outcomes through several pathways, including their influence on health-related behavioural patterns such as smoking, alcohol use or medical help-seeking^(10,11)^. However, limited studies have investigated the relationship between social networks and dietary habits among older adults^(12,13)^.

Dietary variety, defined as the number of different foods or food groups consumed during a given period, is among the significant indicators of a balance of nutrients^(14)^. Higher dietary variety improves the adequacy of nutrient intake^(15)^. The dietary variety score (DVS) was developed by Kumagai *et al*. to evaluate dietary variety among older Japanese adults^(16)^. DVS validity has been adequately confirmed for energy, protein and the intake of some nutrients^(17)^. A low DVS is related to malnutrition, depression, poor sleep quality, lower physical performance and frailty among older adults^(18–25)^. DVS is affected by various factors, such as age, gender, chewing ability, intellectual activity, living arrangements, economic status and food accessibility^(17,25–28)^. A previous study reported that perceived social support for diet, such as having someone to help with food shopping and receiving any food from neighbours or relatives, acted as a buffer against low DVS among older adults who live alone^(27)^. However, the association between social networks and DVS is unclear.

Therefore, this study aimed to examine the association between social networks and DVS among community-dwelling older adults. Since social networks have been suggested to influence health behaviours, we hypothesise that poor social networks lead to deficient dietary variety among older adults. Furthermore, we consider the influence of age, gender and living arrangements on social networks among older adults based on prior related literature. For instance, older adults’ social networks have shown a reverse progressive sequence with an increase in age^(29)^. Furthermore, social network size in old age has been found to expand more for women than men^(30)^. Additionally, lower social network scores have been reported among older adults living alone than those living with someone^(2)^. Therefore, further analyses were conducted to determine the association between social networks and DVS after stratifications based on age, gender and living arrangements.

## Methods

### Participants and study period

This cross-sectional study was conducted using a follow-up survey from our previous study^(25,31)^. The study participants were community-dwelling older people from the overall older population aged ≥ 65 years in N City, H Prefecture, Japan, excluding hospitalised older people and those who resided in a nursing home. The participants included respondents who agreed to receive our follow-up survey at the time of the baseline survey. Of the 2794 participants in the baseline survey, 1635 responded to the prospective survey (58·5 %). The survey forms, including research descriptions such as the aim of this study, were sent to these participants by mail in mid-August 2021. Of the 1635 participants, 1243 participants (76·0 %) responded to the survey questionnaire. However, twenty-four respondents were excluded owing to missing data on age and DVS. Finally, 1229 participants were included in the study.

### Social networks

Social networks were assessed using the Japanese version of the abbreviated Lubben Social Network Scale (LSNS-6)^(2,32)^. The LSNS-6 covers emotional and instrumental support and comprises three items each related to family networks and friendship networks. The number of people in each network was determined on a 6-point scale. The LSNS-6 score ranged from 0 to 30 points; higher scores indicated larger social networks and scores < 12 indicated social isolation.

### Dietary variety

Dietary variety was assessed using the DVS developed for older Japanese people^(16)^. Participants selected the intake frequencies of ten food groups, which included fish and seafood, meat, eggs, dairy products, soyabean and its products, green and yellow vegetables, seaweed, potatoes, fruits and fat or oils, in the last week from the following choices: ‘eat almost every day’, ‘eat once every 2 d’, ‘eat 1 or 2 d a week’ and ‘hardly eat at all’. The choice of ‘eat almost every day’ led to a score of 1, while the other choices led to a score of 0. The sum of the ten food group scores was used to express the DVS. The DVS ranged from 0 to 10 points, with a higher score indicating higher dietary variety. Scores of < 4, 4–6 and ≥ 7 points indicated low, middle and high DVS, respectively. The validity of DVS has been previously confirmed^(17)^.

### Other variables

Demographic data were obtained on variables including comorbidities, living arrangements and subjective economic status. The number of comorbidities was obtained by counting self-reported comorbidities; in particular, self-reported cancer, cardiac disease, stroke, hypertension, diabetes and dyslipidaemia were assessed as comorbidities. Living arrangement was assessed from participants’ responses to whether they lived alone or with someone. Referring to our previous study, subjective economic status was assessed using a five-point scale ranging from very secure to very insecure and was divided into two groups: secure and insecure^(33)^. Secure included the following responses: ‘very secure’, ‘secure’, and ‘neither’; insecure included the following responses: ‘insecure’ and ‘very insecure’. Since DVS is affected by chewing ability, the oral frailty score was assessed using the oral frailty checklist proposed by the Japan Dental Association, to determine oral function^(26,34)^. The oral frailty score was calculated as the sum of points of the eight questions regarding oral functions, and the oral frailty risk was divided into low risk, risk and high risk according to previous reports^(34)^.

### Statistical analysis

The participants were divided into low, middle and high DVS groups. Age was calculated from the date of birth obtained in the baseline survey. Gender details were obtained from the baseline survey data. Participant characteristics, as quantitative variables, were expressed as mean ± sd (median). Categorical variables were expressed as numbers (percentages). The Kruskal–Wallis and chi-squared tests for quantitative and categorical variables, respectively, were used to compare the participants among the three DVS groups. Multivariable linear regression analysis was used to analyse crude, age- and gender-adjusted and multivariable-adjusted correlations between the LSNS-6 score and DVS. The covariates of the multivariable-adjusted model included age, gender (women), number of comorbidities, subjective economic insecurity and oral frailty score (continuous variable). The assumptions of linearity and constant variance of error in the models were checked by scatter plots. The normality in the models was checked by determining whether the residuals have a normal distribution with histograms^(35)^. All models met these assumptions. The association between social isolation and low DVS was tested using logistic regression analysis to further calculate OR and 95 % CI. Covariates of the logistic analysis included the following: ≥ 75 years of age, gender (women), living alone, hypertension, diabetes, subjective economic insecurity and oral frailty risk (low risk, risk and high risk). Variables that showed significant differences in the comparisons among the DVS groups (*P* < 0·05) were chosen as covariates in the linear regression and logistic regression analyses. Living alone was also included as a covariate since a previous study reported an association between this variable and DVS^(25)^. The correlations between the LSNS-6 and DVS were examined through stratified analysis by age, gender and living arrangements, which have been reported to influence social networks^(2,29,30)^. A sensitivity analysis was performed using mean imputation to examine the possible influence of missing data on the results. IBM SPSS Statistics 26.0 (IBM Corp.) was used to analyse all the statistical data. Two-tailed *P* < 0·05 was considered statistically significant.

## Results

### Participant characteristics

Table 1 shows the overall participant characteristics and those based on DVS groups. The average ± sd (median) age of the participants was 75·2 ± 6·0 (74·0) years. Of the total participants, 45·6 % (*n* 561) were men and 54·4 % (*n* 668) were women. The average ± sd of the LSNS-6 score was 13·1 ± 5·6 (13·0); 38·1 % of the participants were considered socially isolated (LSNS-6 score < 12). Populations with cancer, heart disease, stroke, dyslipidaemia and those that lived alone differed insignificantly among the three DVS groups. However, the three DVS groups exhibited significant differences in age, gender, number of comorbidities, population with hypertension and diabetes, subjective economic status, oral frailty score and oral frailty risk. Compared with the low DVS group, the age and population of women were significantly higher in the middle and high DVS groups. In the low DVS group, the number of comorbidities and the economically insecure population were high. Additionally, the oral frailty score and high-risk population with oral frailty in the low DVS group were significantly higher than those in the middle and high DVS groups. The LSNS-6 score among the three DVS groups differed significantly. The low DVS group had a low LSNS-6 score compared to the middle and high DVS groups (12·2 ± 5·6 *v*. 13·4 ± 5·4 and 14·4 ± 5·7, *P* < 0·001). Furthermore, the socially isolated population in the low DVS group was higher than that in the middle and high DVS groups (43·5 % *v*. 35·8 % and 31·0 %, *P* = 0·005).

**Table 1:** Overall participant characteristics and those according to DVS groups (*n* 1229)

	Overall			Low DVS				Middle DVS				High DVS					*P*
Variables	*n*	%	Median	*n*	*n*	%	Median	*n*	*n*	%	Median	*n*	*n*	%	Median	*n*	
Age, year																	
Average	75·2		74·0	1229	74·6		73·0	469	75·5		75·0	589	76·1		75·0	171	0·004†
sd	6·0				6·0				5·9				6·1				
< 75 years	644	52·4			225	58·6			291	49·4			78	45·6			0·002
≥ 75 years	585	47·6			194	41·4			298	50·6			93	54·4			
Gender																	
Men	561	45·6			278	59·3			225	38·2			58	33·9			< 0·001‡
Women	668	54·4			191	40·7			364	61·8			113	66·1			
Number of comorbidities																	
Average	1·5		1·0	1157	1·5		1·0	447	1·5		1·0	551	1·2		1·0	159	0·021†
sd	1·3				1·3				1·3				1·1				
Comorbidities																	
Cancer	67	5·8			24	5·4			30	5·4			13	8·2			0·382‡
Heart diseases	147	12·7			68	15·2			60	10·9			19	11·9			0·119‡
Stroke	24	2·1			10	2·2			11	2·0			3	1·9			0·950‡
Hypertension	425	36·7			183	40·9			206	37·4			36	22·6			< 0·001‡
Diabetes	149	12·9			54	12·1			83	15·1			12	7·5			0·036‡
Dyslipidaemia	149	12·9			45	10·0			81	14·7			23	14·5			0·077‡
Missing				72				22				38				12	
Living arrangements																	
Living with someone	958	77·9			368	78·5			458	77·8			132	77·2			0·932‡
Living alone	271	22·1			101	21·5			131	22·2			39	22·8			
Subjective economic status																	
Secure	1149	40·0			422	90·8			560	95·7			167	98·2			< 0·001‡
Insecure	71	5·8			43	9·2			25	4·3			3	1·8			
Missing				9				4				4				1	
Oral frailty score																	
Average	3·4		3·0	1229	3·7		3·0	469	3·2		3·0	589	3·2		3·0	171	0·007†
sd	2·4				2·5				2·2				2·4				
Oral frailty risk																	
Low risk	509	41·4			174	37·1			256	43·5			79	46·2			0·023‡
Risk	211	17·2			73	15·6			108	18·3			30	17·5			
High risk	509	41·4			222	47·3			225	38·2			62	36·3			
LSNS-6 score																	
Average	13·1		13·0	1229	12·2		12·0	469	13·4		13·0	589	14·4		14·0	171	< 0·001†
sd	5·6				5·6				5·4				5·7				
< 12	468	38·1			204	43·5			211	35·8			53	31·0			0·005‡
≥ 12	761	61·9			265	56·5			378	64·2			118	69·0			
DVS score																	
Average	4·2		4·0	1229	2·1		2·0	469	4·9		5·0	589	7·7		7·0	171	< 0·001†
sd	2·1				0·9				0·8				0·9				

DVS, dietary variety score; LSNS, Lubben Social Network Scale.

† Kruskal–Wallis test.

‡ X^2^ test.

### Associations between the Lubben Social Network Scale-6 score and social isolation with DVS

The association between the LSNS-6 score and DVS was analysed using multivariate linear regression analysis (Table 2). The LSNS-6 score positively correlated with DVS in the crude (standardised coefficient, 0·148; *P* < 0·001) and the age- and gender-adjusted (Model 1; standardised coefficient, 0·132; *P* < 0·001) and multivariate-adjusted models (Model 2; standardised coefficient, 0·108; *P* < 0·01). Model 3, in which the oral frailty score was added as a covariate, displayed a constant significant association (standardised coefficient, 0·092; *P* = 0·002). Furthermore, the association between social isolation and low DVS was examined using logistic analysis (Table 3). Social isolation (LSNS-6 score < 12) was found to be significantly associated with low DVS (score < 4) in the crude (OR, 1·45; 95 % CI 1·14, 1·86; *P* = 0·003) and the age- and gender-adjusted (Model 1; OR, 1·39; 95 % CI 1·08, 1·79; *P* = 0·010) and multivariate models (Model 2; OR, 1·36, 95 % CI 1·05, 1·75; *P* = 0·019). The significant association between social isolation and low DVS also remained constant in Model 3, which included the oral frailty score as a covariate (OR, 1·30; 95 % CI 1·00, 1·68; *P* = 0·048). These results showed that social networks were associated with dietary variety among community-dwelling older adults.

**Table 2:** Liner regression analysis between the DVS and LSNS-6 (*n* 1148)

	Unstandardised coefficients	Unstandardised coefficients 95 % CI	Standardised coefficients	*P*
		Lower, Upper		
Crude				
LSNS-6	0·056	0·034, 0·078	0·148	< 0·001
				
Model 1				
LSNS-6	0·050	0·028, 0·071	0·132	< 0·001
Age	0·032	0·012, 0·052	0·091	0·001
Gender, women	0·815	0·578, 1·052	0·195	< 0·001
				
Model 2				
LSNS-6	0·041	0·019, 0·062	0·108	< 0·001
Age	0·037	0·017, 0·057	0·105	< 0·001
Gender, women	0·831	0·591, 1·072	0·199	< 0·001
Living alone	−0·231	−0·525, 0·049	−0·062	0·122
Number of comorbidities	−0·100	−0·194, −0·006	−0·060	0·037
Subjective economic status, insecure	0·929	−1·428, −0·430	−0·105	< 0·001
				
Model 3				
LSNS-6	0·035	0·013, 0·057	0·092	0·002
Age	0·046	0·025, 0·066	0·131	< 0·001
Gender, women	0·834	0·595, 1·073	0·199	< 0·001
Living alone	−0·252	−0·543, 0·040	−0·050	0·091
Number of comorbidities	−0·083	−0·177, 0·011	−0·050	0·082
Subjective economic status, insecure	−0·841	−1·339, −0·344	−0·095	0·001
Oral frailty score	−0·107	−0·158, −0·055	−0·120	< 0·001

DVS, dietary variety score; LSNS, Lubben Social Network Scale.

**Table 3:** Logistic regression analysis between low DVS (< 4) and social isolation (*n* 1148)

	OR	95 % CI	*P*
		Lower, Upper	
Crude			
Social isolation, LSNS-6 < 12	1·45	1·14, 1·86	0·003
			
Model 1			
Social isolation, LSNS-6 < 12	1·39	1·08, 1·79	0·010
Age ≥ 75 years	0·64	0·50, 0·82	< 0·001
Gender, women	0·40	0·31, 0·51	< 0·001
			
Model 2			
Social isolation, LSNS-6 < 12	1·36	1·05, 1·75	0·019
Age ≥ 75 years	0·69	0·48, 0·80	< 0·001
Gender, women	0·390	0·30, 0·50	< 0·001
Living alone	1·20	0·880, 1·63	0·263
Hypertension	1·31	1·01, 1·69	0·043
Diabetes	0·74	0·51, 1·08	0·117
Subjective economic status, insecure	2·20	1·30, 3·72	0·003
			
Model 3			
Social isolation, LSNS-6 < 12	1·30	1·00, 1·68	0·048
Age ≥ 75 years	0·56	0·43, 0·73	< 0·001
Gender, women	0·39	0·30, 0·50	< 0·001
Living alone	1·21	0·88, 1·66	0·237
Hypertension	1·27	0·98, 1·65	0·069
Diabetes	0·70	0·48, 1·03	0·071
Subjective economic status, insecure	2·07	1·22, 3·51	0·007
Oral frailty risk	1·31	1·13, 1·51	< 0·001

DVS, dietary variety score; LSNS, Lubben Social Network Scale.

### Stratified analysis of the association between the Lubben Social Network Scale-6 and dietary variety score using liner regression analysis

The association between the LSNS-6 and DVS was stratified by age, gender and living arrangement (Table 4). In the multivariate-adjusted models, the results show that the LSNS-6 and DVS were significantly associated in participants of a younger age (age < 75 years; standardised coefficient, 0·122; *P* = 0·001), women respondents (standardised coefficient, 0·103; *P* = 0·011) and those living with someone (standardised coefficient, 0·085; *P* = 0·010). In contrast, the LSNS-6 scores were insignificantly related to DVS in participants with the following characteristics: older age (≥ 75 years), men and living alone.

**Table 4:** Stratified analysis of the association between the LSNS-6 and DVS using liner regression analysis (*n* 1148)

	Unstandardised coefficients	Unstandardised coefficients 95 % CI	Standardised coefficients	*P*
		Lower, Upper		
Age < 75 years (*n* 611)				
LSNS-6*	0·052	0·023, 0·081	0·122	0·001
Age ≥ 75 years (*n* 537)				
LSNS-6*	0·016	−0·016, 0·049	0·044	0·322
				
Men (*n* 532)				
LSNS-6†	0·029	−0·002, 0·060	0·080	0·069
Women (*n* 616)				
LSNS-6†	0·039	0·009, 0·070	0·103	0·011
				
Living with someone (*n* 898)				
LSNS-6‡	0·032	0·008, 0·057	0·085	0·010
Living alone (*n* 250)				
LSNS-6‡	0·041	−0·004, 0·086	0·110	0·075

DVS, dietary variety score; LSNS, Lubben Social Network Scale.

* Adjusted for age, gender, number of comorbidities, living arrangements, subjective economic status and oral frailty score.

† Adjusted for age, number of comorbidities, living arrangements, subjective economic status and oral frailty score.

‡ Adjusted for age, gender, number of comorbidities, subjective economic status and oral frailty score.

### Sensitivity analysis

Mean imputations were conducted to account for missing data on the number of diseases requiring therapy, hypertension, diabetes and subjective economic status. The mean imputation results ascertained similar relationships between the LSNS-6 and social isolation with DVS.

## Discussion

The results of this study revealed social networks to be positively associated with dietary variety among randomly selected community-dwelling older adults. Additionally, social isolation showed a significant relationship with low dietary variety, indicating low dietary quality. These associations were found to be independent of age, gender, living arrangements, economic insecurity and oral frailty. Significant associations between social network and dietary variety were observed among those aged < 75 years, women and those living with someone, rather than among those aged ≥ 75 years, men and those living alone. Overall, poor social networks may lead to worse dietary variety among community-dwelling older adults. Moreover, the association between social networks and dietary variety was found to be influenced by age, gender and living arrangements. To the best of our knowledge, the association between social isolation and dietary variety has not been described before. A low DVS causes malnutrition, depression, lower physical performance and frailty among older adults^(18,19,22–24)^. Accordingly, our results, which show the importance of social networks for DVS, have implications for supporting healthy ageing.

Several studies support our results. Kwon *et al*. reported that loss of a spouse is a significant predictor of the decline in DVS^(26)^. Frequent friend contact is associated with a better variety of vegetables among older adults who are widowed or have limited family contact^(36)^. Although the mechanisms of the association between social networks and dietary variety were unclear in this study, Kurimoto *et al*. showed that larger social networks facilitated access to social support among older adults^(2)^. A related qualitative study^(37)^ notes that the limited use of social support for grocery shopping and cooking is among the determinants of eating behaviours among older adults, while food-related social support availability, such as help with grocery shopping and/or food exchange, was observed as a related factor for DVS^(27,37)^. Moreover, several mechanisms, such as social influence/social comparison, social control, role-based purpose and meaning, self-esteem, sense of control, belonging and companionship, might be related to the association between social networks and dietary behaviour^(38)^.

Differences were observed in the association between social networks and DVS according to age, gender and living arrangement. This study notes an association between social networks and DVS among oldest-old adults rather than young-older adults. According to the multivariate linear regression analysis, the number of comorbidities was significantly associated with DVS among the oldest-old adults (standardised coefficient, –0·107; *P* = 0·012), whereas this association was not observed among young-old adults (standardised coefficient, 0·005; *P* = 0·890). The number of comorbidities among oldest-old and young-old adults was 1·7 ± 1·3 (1·0) and 1·3 ± 1·2 (1·0), respectively (*P* < 0·001, Mann–Whitney U test). Several diseases, including hypertension, have been reported to be associated with reduced DVS^(39)^. The present results also indicated that the hypertension and diabetes rates in the low DVS group were higher than those in the high DVS group (Table 1). The association between social networks and DVS among the oldest-old adults may have weakened owing to an increase in diseases that affect eating habits. The gender differences in the influence of social networks on older adults’ diets are consistent with those of other studies^(27,40)^. One of the pathways through which social networks influence health behaviours is the provision of social support. A previous study reported that the association between social support and vegetable and fruit intake was found only in women and not in men, suggesting that the social aspects of meals are crucial for maintaining an adequate diet among women^(41)^. A possible reason for the gender difference in the association between social ties and health behaviours is that women tend to maintain more emotionally intimate relationships than men^(11)^. Therefore, women may be more likely to receive support from the members of their social networks than men. In addition, a qualitative study also reported that the role of family and friends in motivating women is particularly important^(42)^. Hence, this study observes an association between social networks and dietary variety among women rather than men. Regarding living arrangements, our results were inconsistent with those of a previous study. This study shows that social networks and DVS are associated in those living with someone, while Conklin *et al*. reported that frequent contact with friends increases vegetable variety among older adults who live alone. However, no association was found among those who live with others^(36)^. Our previous study showed that the difference in living arrangements in relation to the DVS was influenced by gender^(25)^. Differences in living arrangements in the association between social networks and DVS might be complicated owing to the involvement of multiple factors. Further research must include other factors related to living alone, such as the period of living alone and life events that lead to living alone (e.g. divorce, death of spouse or children).

The association between social networks and dietary variety was adjusted by the oral frailty score in this study, as chewing ability is a critical influencing physical factor for eating behaviour among older adults^(26,43)^. The independent relationship between social networks and dietary variety was determined in our multivariate analysis, which included the oral frailty score as a covariate. This result suggests that physical and social care are important for dietary management among older adults.

The strengths of this study are as follows. First, to the best of our knowledge, it was the first to identify an association between social networks and DVS among older adults. Second, it involved stratified analysis with almost comparable age and gender ratios. However, the number of participants living alone was smaller than the number of participants living with someone; thus, the statistical power was small in the living alone group. This study had a few other limitations. First, it was a cross-sectional study. The results need to be interpreted with caution since we could not discuss causal relationships. Second, 58·5 % of the respondents accepted the follow-up study at the baseline survey. Additionally, the response rate in the follow-up survey was 76·0 %. This might have resulted in sampling bias and reduced generalisability. In the baseline survey, while no differences were observed in age, number of diseases or percentage living alone according to the presence or absence of acceptance for the follow-up survey, men had a higher percentage of acceptance than women did. The DVS was significantly higher for those who accepted compared to those who did not (data not shown). The study population may have been biased towards those with a high level of health awareness. Third, all variables were self-reported data obtained from the mail survey. Thus, a social desirability response bias may affect the DVS and the social network scores in this study. Fourth, the survey was conducted during the COVID-19 pandemic when almost no community activities intended for older adults occurred. This may have influenced the self-evaluation of the social networks. Fifth, we did not assess quantitative food or nutrient intake, whereas the DVS was validated to relate to nutrient intake^(17)^. Finally, although depression is a key influencing factor of dietary variety, we could not assess such psychological conditions in this study^(19)^.

In conclusion, this study found that poor social networks and social isolation are related to reduced dietary variety among community-dwelling older adults. Particularly, the association between social networks and dietary variety was shown in the following subgroups: young-old adults, women and those living with someone. These results suggest that screening older adults who have poor social networks and increased social support may contribute to improved food variety and health. However, further research is needed to identify effective social support for maintaining healthy dietary behaviour among socially isolated older adults.
